# Comparative transcriptomic and weighted gene co-expression network analysis to identify the core genes in the cultivars of *Musa acuminata* under both infected and chemical perturbated conditions

**DOI:** 10.1080/15592324.2023.2269675

**Published:** 2023-11-10

**Authors:** PTV Lakshmi, Amrendra Kumar, Ajna A. S., Abitha P Raveendran, Anjali Chaudhary, Adhitthan Shanmugam, Annamalai Arunachalam

**Affiliations:** a Phytomatics Lab, Department of Bioinformatics, School of Life Sciences, Pondicherry University, Pondicherry, India; b Department of Food Science and Technology, School of Life Sciences, Pondicherry University, Pondicherry, India

**Keywords:** Banana, DEGs, *Fusarium oxysporum f. sp. cubense TR4*, transcriptomics, WGCNA

## Abstract

Banana is a high nutrient crop, which ranks fourth in terms of gross value production. Fusarium wilt of banana, caused by *Fusarium oxysporum f. sp. cubense* tropical race 4 (FocTR4), is considered the most destructive disease leading to the complete loss of production of the Cavendish cultivars Berangan, Brazilian and Williams, which are vulnerable to the infection of FocTR4. However, the treatment with benzothiadiazole, a synthetic salicylic analog, is aimed to induce resistance in plants. Thus, the treatments pertaining to the banana plants subjected to the Foc infection within the chosen cultivars were compared with chemically treated samples obtained at different time intervals for a short duration (0–4 days). The integrated omics analyses considering the parameters of WGCNA, functional annotation, and protein–protein interactions revealed that many pathways have been negatively influenced in Cavendish bananas under FocTR4 infections and the number of genes influenced also increased over time in Williams cultivar. Furthermore, elevation in immune response and resistance genes were also observed in the roots of the Cavendish banana.

## 1. Introduction

Banana (*Musa acuminata*) of Musaceae family, commonly known as the ‘Apple of paradise’, is the oldest fruit known to mankind (ICAR, https://kvk.icar.gov.in/API/Content/PPupload/k0331_41.pdf)^1^ and is considered as one of the most valuable primary agricultural commodities, which is consumed raw, fried, or brewed. According to FAO (https://www.fao.org/markets-and-trade/commodities/bananas/en/), banana is one of the most produced, traded, and consumed fruit worldwide and is cultivated in over 150 countries where it is believed that this fruit originated in South Asia and was the first cultivated crop.^2^ Between 2000 and 2019 alone, global fruit production of banana and plantains hold the highest global share of 8% among watermelons, apples, oranges, and grapes, which in total has increased by 54% from 311 million tons to 883 million tons (FAO. 2021. *World Food and Agriculture – Statistical Yearbook 2021*. Rome https://doi.org/10.4060/cb4477en). Over a thousand different varieties of bananas are grown worldwide, but the Cavendish variety is regarded as one of the most significant commercial variants, *M. acuminata* and *Musa balbisiana* carrying genomes A and B, respectively, however is triploid containing the genome AAA for Cavendish^3^ while has the copy number of the genome AAB for plantains.^4^ Bananas have high crude protein content than plantains with 0.48%, and also the total energy value was found to be high in banana (310.55 ± 0.01%) when compared to plantain (273.44 ± 0.01%), which makes banana a more significant fruit crop.^5^

Banana belongs to the Domain Eukaryota; Kingdom Plantae; Phylum Spermatophyta; Subphylum Angiospermae; Class Monocotyledonae; Order Zingiberales; Family *Musaceae*; Genus Musa, and Species *Musa acuminata*. Cultivar group AAA has three Cultivar varieties namely Berangan,^6^ Brazilian,^6^ and Williams.^7^ Being nutritious, it is considered to contain numerous enzymes, antioxidants like phenolics, flavonoids, dopamine, etc.^8^ The high antioxidant properties of banana are responsible for good digestion compared to other fruits. Furthermore, it acts as a great source of dietary fiber, vitamin C, vitamin B6, potassium (K), and manganese (Mn) with high calorie count,^9^ which can lower the risk of various chronic degenerative diseases.

Considering the importance of Cavendish banana, this study aims to investigate the role of fungal and chemical perturbations on the Cavendish subgroups Brazilian, Berangan, and Williams. Brazilian, a dwarf banana known as Santa Catarina Silver, belongs to Brazil and is sweet and plumpy in nature; Berangan or Pisang Berangan is a medium-sized banana that belongs to West Malaysia with a balanced sweet and sour flavor; and Williams is a giant cultivar (https://www.promusa.org/).

Fusarium wilt of banana is a severe and fatal fungal disease caused by the soil-borne pathogen, *Fusarium oxysporum f. sp. cubense* (Foc) that threatens 15% of worldwide banana production and nearly half of the world’s total banana cultivated area.^10,11^ *F. oxysporum*-infected xylem vessels turn reddish-brown^12^ and eventually get clogged, hindering the translocation of water and nutrients to the upper parts of the plant. During the infection, older leaves begin to wither, die, and split at the base. Yellowing of the leaf lamina is typical, owing to the pathogen’s production of phytotoxins, while non-yellowing development of wilt symptoms is also documented; the infection extends to younger leaves, and ultimately, terminates plant growth.^2^

*Fusarium oxysporum f. sp. cubense* has been divided into four physiological races, namely race 1, race 2, race 3, and race 4 that infect banana. *F. oxysporum f. sp. cubense* race 4 (FocR4) again has two biotypes that are *F. oxysporum f. sp. cubense* tropical race 4 (FocTR4) and *F. oxysporum f. sp. cubense* subtropical race 4 (FocSTR4). Each race is specific to its host organism. Race 1 infected banana cultivars like “Gros Michel” (*Musa* sp. AAA genome), “Pome,” “silk,” and “Pisang Awak” (*Musa* sp. AAB group) are responsible for the 20^th^ century epidemic,^13^ while race 2 infects the cultivar “Bluggoe” and its closely related cultivars. Race 3 does not contaminate any of the *Musa* species; on the contrary, race 4 has a broad host range, infecting almost all cultivars, including “Dwarf Cavendish” (*Musa* sp. AAA genome), as well as the hosts of race 1 and race 2.^14^ Hence, race 4 is considered the most prevalent and devastating biotype of Foc in tropical area that causes Panama (Fusarium wilt) disease in bananas, and its occurrence is affecting up to 50% yield in Bihar and Uttar Pradesh states of India.^11^

However, the identification of novel cultivars through resistant breeding is one method to overcome this; this effort is frequently hampered by triploid bananas that limited seed production. Another option is to use nontoxic plant resistance inducers (PRIs), which can help plants to fight pathogens by stimulating their defense mechanisms. Exogenous compounds such as beta amino butyric acid, hexanoic acid, and benzothiadiazole (BTH) have been found to be effective against the pathogen in plant species.^15–17^ Although comparative studies between FocSTR4-affected and normal Cavendish banana group cultivars have revealed the critical roles of pathogenesis-related proteins,^18^ signaling, cell wall lignification, and hypersensitive response in host resistance against FocTR4, these comparisons seemed to provide a scope to determine the resistant mechanism. Accordingly, the role of BTH in enhancing banana plant defense response to FocTR4 infection^19^ has also revealed that BTH selectively affects the biological processes (BPs) associated with plant defense. They also observed several genes that were up- and downregulated in the roots and leaves. Furthermore, it was observed that being a synthetic salicylic acid analog, BTH was able to induce an effect during the host–pathogen interaction as genes responsible for plant defense like receptor-like kinases were more positively active in Foc-infected samples than BTH unsprayed plants.^20^ A number of systemic acquired resistance-associated genes were activated when BTH was exogenously applied to the leaves of wheat and Arabidopsis, leading to enhanced plant protection against various pathogens.^21^

However, no comparative study of a cultivar with induced resistance due to BTH application and a susceptible cultivar of FocTR4 has been published. Therefore, an investigation in this direction would certainly help us to understand the genes responsible for enhancing the growth of BTH- treated conditions under pathogen-induced stress condition in plants. This would perhaps provide knowledge on how BTH levels affect banana against FocTR4. Thus, to understand the mechanism behind the infection process and the effect of BTH treatment, studies were undertaken to compare the three different cultivars (Berangan, Brazilian, and Williams) under both normal (control) and fungal infectious conditions, which included the examination of differential expression gene pattern existing within and between the cultivars.

## 2. Materials and Methods

### 2.1. Dataset collection

Multiple dataset of samples for the different cultivars were retrieved from the National Centre for Biotechnology Information – Sequence Read Archive (NCBI SRA), a public domain database. BioProject accession numbers PRJNA417328,^19^ PRJNA287860^6^ (https://www.ncbi.nlm.nih.gov/bioproject/PRJNA287860), PRJNA322439 (https://www.ncbi.nlm.nih.gov/bioproject/?term=PRJNA322439), PRJNA319058^22^ were selected for Berangan, Brazilian, and William cultivars, respectively, whose roots were infected with FocTR4 fungi and subjected to BTH treatment at different intervals **(Table S1A**). The paired-end RNA-Seq raw reads of these cultivars were also collected from banana plant root samples.

### 2.2. Data preprocessing and alignment

**Figure 1.**
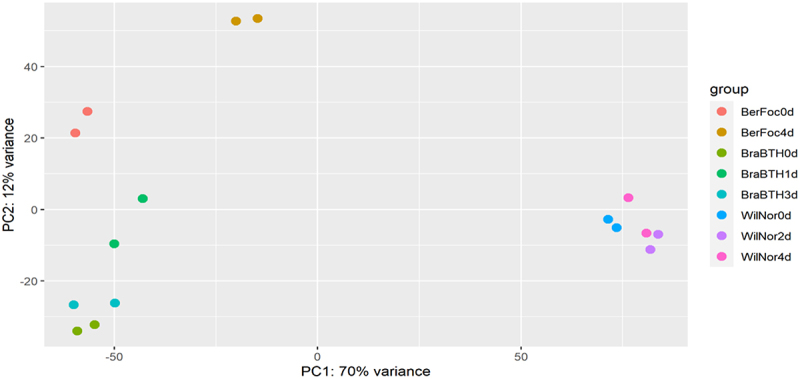
Exploration of samples of the cultivars obtained during different time intervals. Principal component analysis (PCA) plot indicates the distribution of the data based on the distances and helps in identifying the data for perfect correlation. BerFoc0d: control sample of Berangan; BerFoc4d: FocTR4, 4 days treated; BraBTH0d: FocTR4, 0 day treated; BraBTH1d: FocTR4, 1 day treated; BraBTH3d: FocTR4 3 days treated; WilNor0d: control sample of Williams cultivar; WilNor2d: 2-day-old normal sample of Williams cultivar; WilNor4d: 4-day-old normal sample of Williams cultivar.

The quality of all four datasets were computed using the FASTQC tool(http://www.bioinformatics.babraham.ac.uk/projects/fastqc/), and the raw read sequences of the datasets were mapped to the latest reference sequence *M. acuminata* (v2) using the HISAT2 tool.^23^ The read count of each gene mapped to the reference genome was calculated using the FeatureCounts tool.^24^ The different datasets have different sampling time intervals ranging from 0, 24, 48, 72, and 96 h and obtained as replicate as represented in Figure 1.

### 2.3. Identification of common genes in different combinations of DEGs

Differential expression analysis was performed pairwise for BerFoc0d vs. BerFoc4d (BerFoc0vs4d, Berangan cultivar infected with FocTR4 on the day it was inoculated and on the fourth day after inoculation); BraBTH0d vs. BraBTH1d (BraBTH0vs1d, Brazilian cultivar at the day it was inoculated with chemical moiety, BTH, and 1 day after the treatment); BraBTH0d vs. BraBTH3d (BraBTH0vs3d, Brazilian cultivar at the day it was inoculated with chemical moiety, BTH, and 3 days after the treatment); WilNor0d vs. WilNor2d (WilNor0vs2d, William cultivar with normal growth conditions that is without infection by the Foc and the treatment at the initial day of growth and after 2 days); WilNor0d vs. WilNor4d (WilNor0vs4d, William cultivar with normal growth conditions that is without infection by the Foc and the treatment at the initial day of growth and after 4 days) (**Table S1B**) using DESeq2 of Bioconductor R package,^25^ where differential expression calculation and significant differentially expressed genes (DEGs) were identified by applying the cutoff value of log2 fold change of ≥|1.5| with padj cutoff <0.05. These paired combinations were analyzed to study the behavioral pattern of genes expressed under different conditions at varied time intervals between and within the cultivars of the Cavendish variety of banana.

### 2.4. Weight gene co-expression network analysis

WGCNA tool was used to analyze the co-expression network and Topological Overlap Matrix (TOM). Weight gene co-expression network was constructed to determine the relationship between genes by calculating the similarity matrix among the genes.^26^ To obtain a scale-free network, the type of the network and soft-threshold power 12 have been chosen. Then, TOM was constructed followed by module identification by hierarchical clustering and dendrograms using the matrix values.^27^ The dynamic tree cut identified the modules, and module eigengenes were identified further to summarize the expression patterns of the module’s gene across the sample.^28^ Subsequently, downstream analyses were narrowed down for certain clusters.

### 2.5. Identified core genes in statistically significant positive and negative co-expressed genes

Correlation between module eigengenes and expression of genes were analyzed among the significantly correlated modules of interest associated with root samples of banana. The heatmap was used to represent the correlation values. The module membership (MM) is the association between each module eigengene and its gene expression as gene significance (GS) is defined as the correlation between each trait and its significance in contributing to that specific trait.^29^ The MM and GS (MM > 0.8 closely and GS > 0.5) were determined to identify the key genes in a module^30^ for recognizing the core genes between the significant DEGs.^31^

#### 2.6. Functional annotation

The biological information of significant genes were identified by DEGs and Weighted Gene Co-expression Network Analysis (WGCNA). Gene Ontology (GO) annotations were obtained using the following categories: Biological process (BP), Molecular function (MF), and Cellular component (CC) with a cutoff at FDR=0.05 . Then, a hierarchical tree summarizing the correlation among the 40 most significantly enriched pathways was also generated using ShinyGO v 0.75,^32^ a tool with statistical-based analysis and visualization results with the R package, built using the Ensembl annotation database and numerous other source databases.

### 2.7. PPI network analysis

The retrieval of the interacting proteins from the STRING^33^ database was used to examine the core genes conversion into proteins. The core analysis function was used to interpret the identified proteins in the context of biological functions and pathways in the inventive pathway analysis of the identified proteins. Then, the significant modules and hub genes of pathways were identified using Cytoscape^34^ software and ShinyGO tool.^32^ Furthermore, the MapMan program was used to generate a graphical representation of the core genes involved in biotic stress response pathways^12^. The input command of the protein (converted core genes) are given in the MapMan package to design a particular BP using the banana annotation.

## 3. Results

To understand the mechanism behind BTH-treated and untreated samples, a comparative study of the transcriptome data of *M. acuminata* sub-cultivars was carried out. The DEGs analysis and WGCNA-identified genes were involved in the BTH treatment of *F*FocR4-infected samples. The different datasets were obtained at different sampling time intervals ranging from 0, 24, 48, 72, to 96 h and clustered based on PCA for comparing the influence of both infected and treated samples in terms of expression analysis (Figure 1) and visualize the variation between the samples.

### 3.1 DEGs in multiple cultivars

Three different Cavendish banana cultivars were used to analyze the DEGs at different time periods. Approximately 56.3% of genes (19,835 of 35,275 genes) were found to be significantly differentially expressed in this analysis as compared to the data available with draft genome (Table S2). In the comparative DEGs analysis, 28.04% were expressed in Berangan cultivar 4-day FocTR4-infected samples, 15.9% genes were expressed in Brazilian cultivar within 1-day FocTR4-infected and BTH-treated samples, 1.1% genes were expressed in Brazilian cultivar within 3-day FocTR4-infected and BTH-treated samples, 4.2% genes expressed in 2-day-old Williams cultivar, and 7.2% genes were expressed in 4-day-old Williams cultivar (Table 1).

**Table 1.** DEGs of Cavendish cultivars.

Samples	Downregulated	Upregulated	Total DEG	Total % (Total DEG/Total genes)
BerFoc4d	4353	5535	9888	28.04
BraBTH1d	1595	3980	5575	15.9
BraBTH3d	64	331	395	1.1
WilNor2d	656	813	1469	4.2
WilNor4d	1215	1293	2508	7.2
Total	7883	11,952	19,835	56.3

BerFoc4d: FocTR4, 4 days treated; BraBTH1d: FocTR4, 1 day treated; BraBTH3d: FocTR4, 3 days treated; WilNor2d: 2-day-old normal sample of Williams cultivar; WilNor4d: 2-day-old normal sample of Williams cultivar.

**Figure 2.**
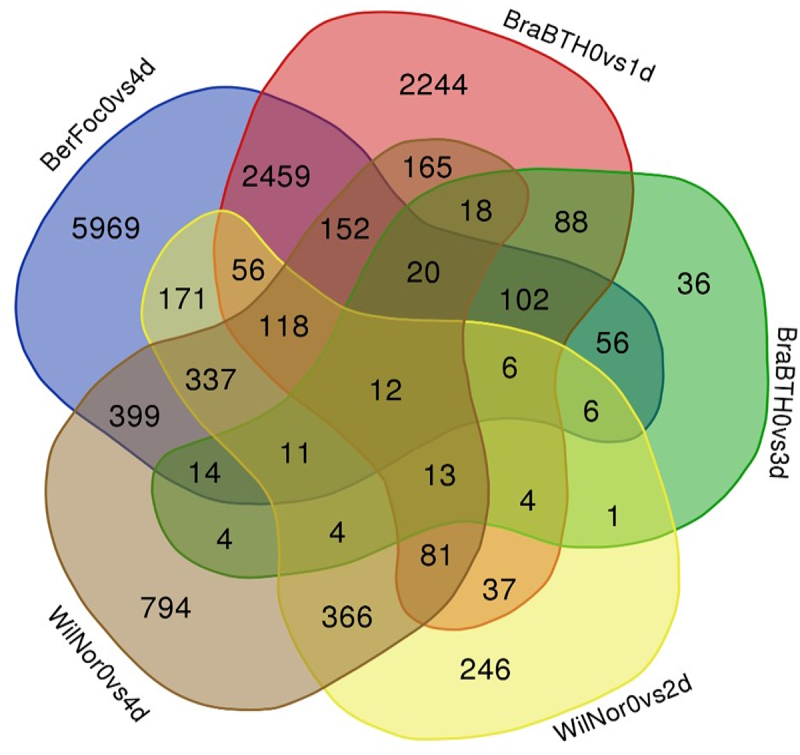
DEG Venn diagram for common genes. The sum of the numbers in the Venn diagram represents the total number of DEGs. The amount of DEGs is shown by the overlap. BerFoc0d: control sample of Berangan; BerFoc4d: FocTR4, 4 days treated; BraBTH0d: FocTR4, 0 day treated; BraBTH1d: FocTR4, 1 day treated; BraBTH3d: FocTR4, 3 days treated; WilNor0d: control sample of Williams cultivar; WilNor2d: 2-day-old normal sample of Williams cultivar; WilNor4d: 4-day-old normal sample of Williams cultivar.

Furthermore, a detailed comparative analysis of the total (13,989) DEGs between the three cultivars (Berangan, Brazilian, and William) was performed. In total, 12 (0.09%) DEGs were expressed in all samples. However, 168 (1.3%) DEGs were intersectionally expressed in four conditions, while 774 (5.6%) DEGs were found to be expressed within the three conditions of cultivars. About 3749 (26.8%) DEGs were expressed across two conditions, and 5969, 2244, 36, 794, and 246 (66. 5%) DEGs were expressed in Berangan cultivar within 4-day-infected FocTR4, Brazilian cultivar within 2-day-infected FocTR4 with BTH-treated samples, Brazilian cultivar within 3-day-infected FocTR4 with BTH-treated samples, 4-day-old Williams cultivar, and 2-day-old Williams cultivar, respectively (Figure 2).

### 3.2. Intersectional co-expression genes analysis

**Figure 3.**
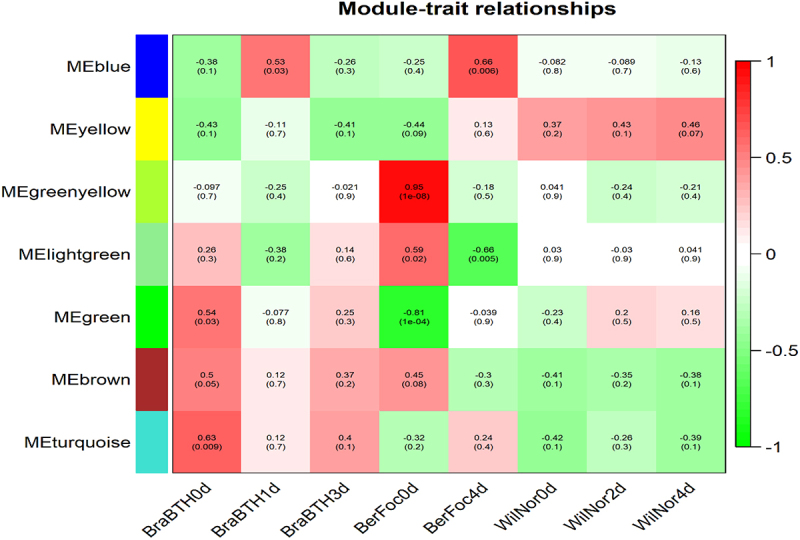
WGCNA to interpret correlation coefficient between modules and samples. Red to green colors depict levels of positive and negative regulation of the genes, respectively. BerFoc0d: control sample of Berangan; BerFoc4d: FocTR4, 4 days treated; BraBTH0d: FocTR4, 0 day treated; BraBTH1d: FocTR4, 1 day treated; BraBTH3d: FocTR4, 3 days treated; WilNor0d: control sample of Williams cultivar; WilNor2d: 2-day-old normal sample of Williams cultivar; WilNor4d: 4-day old normal sample of Williams cultivar.

The total read counts matrix indicated approximately 13,989 genes to be expressed across eight samples (in replicates). The weighted gene co-expression network analysis resulted in seven modules, of which 1774 genes were positively co-expressed in four samples (traits) encompassing five modules (blue, green, greenyellow, lightgreen, and turquoise), whereas 539 genes were negatively co-expressed in two samples represented by two modules (green and lightgreen) (Figure 3; Table S3).

MM and GS measures were calculated to find the functional importance of modules with a high correlation coefficient with samples. The GS measures a gene’s biological relevance, and the MM is the gene’s essentiality in that module.^26^ Apparently, the correlations of genes and modules of both positive and negative co-expressed genes were filtered based on the GS > |0.5| and MM > |0.8|, respectively,^31^ permitted us to compare. The up- and downregulated in modules of blue, green, greenyellow, lightgreen, and turquoise were confirmed to be represented in the upregulated condition emphasizing high stability, 1110 genes experiencing green and lightgreen modules were downregulated and 671 were upregulated genes in BerFoc4d, while in BraBTH1d, 171 genes were upregulated and 125 genes were expressed downregulated (Table 2) in the root (Table S4) samples of banana.

**Table 2.** Identification of the core genes within the co-expressed data.

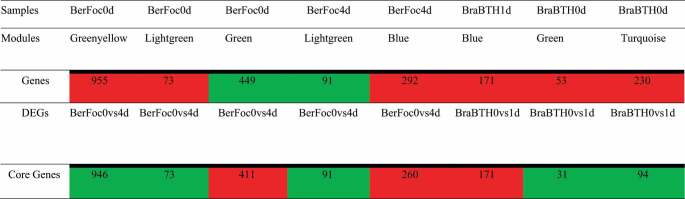	

Red-labeled: positive co-expression; Green-labeled: negative co-expression; Red-labeled: upregulated and Green-labeled: downregulated, based on the DEGs; Core Genes: Common between WGCNA and DEGs; BerFoc0d: control sample of Berangan; BerFoc4d: FocTR4, 4 days treated; BraBTH0d: FocTR4, 0 day treated; 640 BraBTH1d: FocTR4, 1 day treated.

#### 3.3. Functional annotations of DEGs

**Figure 4.**
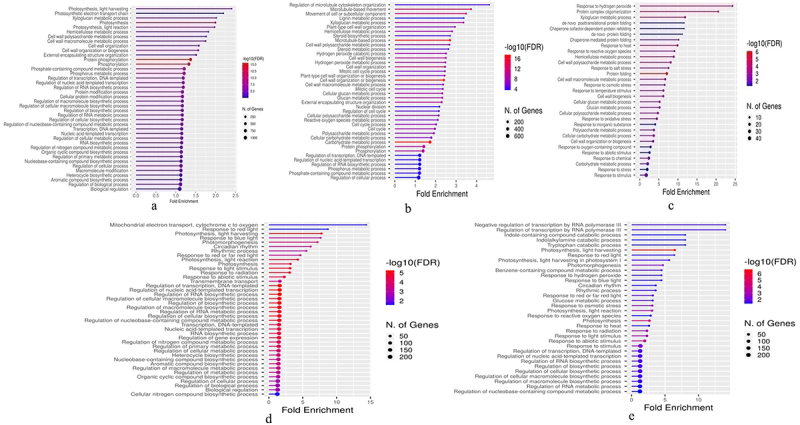
The top 40 BPs of DEGs. a BerFoc04d, b BraBTH01d, c BraBTH03d, d WilNor02d, and e WilNor04d were analyzed for functional enrichment using ShinyGO. Fold enrichment is the ratio of the percentage of genes in a list that belongs to a pathway to the comparable percentage in the background.

Functional annotations of 19,835 DEGs revealed 418 high-level GO categories. Amid which, 25.2% genes were involved in BPs, while 6.3% genes represented CC. Furthermore, 26.9% genes have been encoding for MFs, and 3.5% were associated in the pathways (Table 3; Table S4). In BerFoc0vs4d samples, the genes encoding for the action of protein phosphorylation, phosphorus metabolism, photosynthesis, cell wall organizations, and overall regulations were highly represented (Figure 4a). However, in BraBTH0vs1d samples, the genes associated with microtubule-based movement/process, cell wall biogenesis/organizations, and hydrogen peroxide catabolic/metabolic process have been highly expressed during the last day of infection (Figure 4b), whereas the genes responsible for protein folding, response to stress, stimulus, xyloglucan metabolic process, response to hydrogen peroxide, and cell wall polysaccharide metabolism were highly expressed in BraBTH0vs3d (Figure 4c). In the WilNor0vs2d sample, many genes were involved in BPs, including the regulation of the biosynthetic process, regulation of RNA biosynthetic process, photosynthesis, response to red or far red light, and response to abiotic stress (Figure 4d). Likewise, the 4-day-treated and normally grown samples obviously exhibited all the BPs-associated genes. During 2-day treatment indirectly highly represented their essential role in regulating the plant growth. Herein, in addition to the above, Williams cultivar had many genes that participated in the different BPs such as photomorphogenesis, photosynthesis, light harvesting, response to osmotic stress, tryptophan catabolic process, response to heat, and benzene-containing compound metabolism (Figure 4e; Table S5).

**Table 3.** Enrichment of gene sets by ShinyGO tool.

			High-level		High-level		High-level		High-level
	Significant		GO		GO		GO		GO
Sample	DEGs genes	Genes	category	Genes	category	Genes	category	Genes	category
BerFoc0vs4d	9888	2550	40	727	35	4123	40	394	3
BraBTH0vs1d	5575	1555	40	421	36	217	40	195	5
BraBTH0vs3d	395	75	31	14	4	136	26	10	1
WilNor0vs2d	1469	366	40	8	2	388	10	80	3
WilNor0vs4d	2508	433	34	69	15	458	12	6	1

BerFoc0d: control sample of Berangan; BerFoc4d: FocTR4, 4 days treated; BraBTH1d: FocTR4, 1 day treated; BraBTH3d: FocTR4, 3 days treated; WilNor2d: 2-day-old normal sample of Williams cultivar; WilNor4d: 4-day-old normal sample of Williams cultivar.

### 3.4. PPI interactions and pathways analysis of core genes

**Figure 5.**
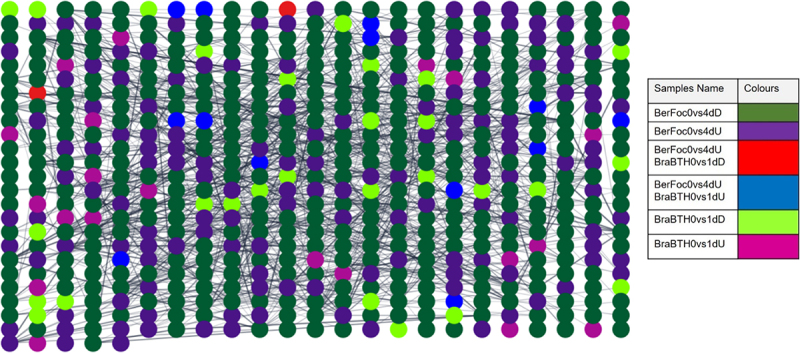
Protein–protein interactions (PPIs) of core genes. Each dot here represents a protein. BerFoc0d: control sample of Berangan; BerFoc4d: FocTR4, 4 days treated; BraBTH0d: FocTR4, 0 day treated; BraBTH1d: FocTR4, 1 day treated; BraBTH3d: FocTR4, 3 days treated; WilNor0d: control sample of Williams cultivar; WilNor2d: 2-day-old normal sample of Williams cultivar; WilNor4d: 4-day-old normal sample of Williams cultivar. U-upregulated; D-downregulated.

**Figure 6.**
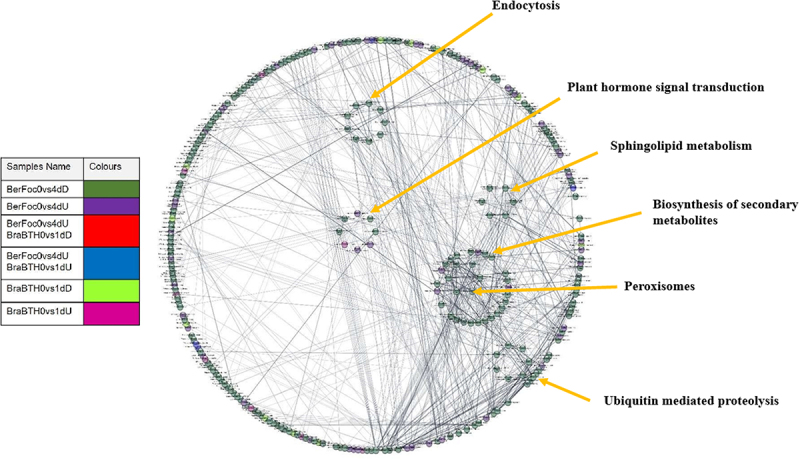
Pathway analysis of core genes. BerFoc0d: control sample of Berangan; BerFoc4d: FocTR4, 4 days treated; BraBTH0d: FocTR4, 0 day treated; BraBTH1d: FocTR4, 1 day treated; BraBTH3d: FocTR4, 3 days treated; WilNor0d: control sample of Williams cultivar; WilNor2d: 2-day-old normal sample of Williams cultivar; WilNor4d: 4-day-old normal sample of Williams cultivar.

**Figure 7.**
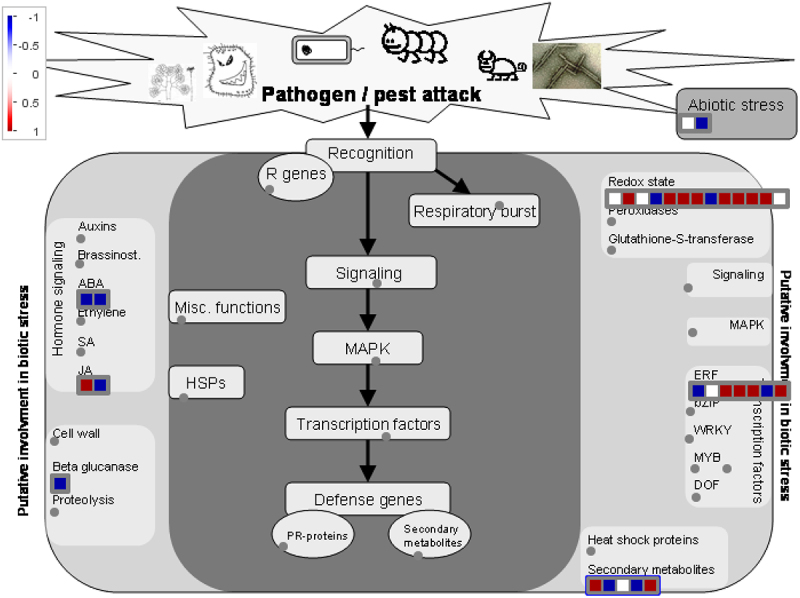
MapMan is a program that visualizes the major genes involved in host–pathogen interactions in FocTR4-treated sample vs. control; the gene implicated in the biotic stress pathway is shown by a color signal, with red representing upregulation and blue suggesting downregulation.

Candidate hub gene identification was performed by considering both MM and GS and DEGs, respectively. Approximately 2077 core genes were recognized (Table 2); from which, 351 protein/string ids were extracted by the involvement of 1110 genes that were downregulated. Alongside, 158 string ids existing in 671 upregulated genes in BerFoc0vs4d conditions were recognized, while 34 string ids from 171 upregulated genes and 30 string ids encoded by 125 downregulated genes were recognized under BraBTH0vs1d conditions. However, within BerFoc0vs4dU and BraBTH0vs1dU (blue color), 13 proteins were observed to be common and upregulated. However, under the same condition, two proteins seemed to be downregulated (red color) especially in BraBTH0vs1dD. Moreover, approximately 21 proteins were uniquely upregulated under BraBTH0vs1dU (pink color), while around 28 proteins were significantly downregulated in BraBTH0vs1dD (bright green). Meanwhile, with the progression of days, approximately 142 and 351 proteins were observed to be up- and downregulated under each BerFoc0vs4dU (violet color) and BerFoc0vs4dD (green color), respectively (Figure 5; Table S6I). Furthermore, in pathway analysis, 20 pathways were identified under significant enrichment FDR, and it was found that many proteins were involved in different pathways including sphingolipid metabolism, peroxisome, ubiquitin-mediated proteolysis, endocytosis, biosynthesis of secondary metabolites, plant hormone signal transduction, etc. (Figure 6; Table S6P). Furthermore, input of core proteins was given to MapMan package, and 26 bin (term) names were identified that contain functional information of the core genes. After that, we identified 26 genes that were classified into the following seven groups: ABA, JA, Beta glucanase, Redox state, ERF, secondary metabolites, and abiotic stress (Figure 7). Most of the core genes in the cell wall organization were under redox state, multi-process regulations in ERF transcription factor. The core genes with known functions, e.g., TFs, redox state, JA, secondary metabolites, and hormone signaling, are shown in Figure 7, and the detailed data are provided in (Table S7). Briefly, most of the core genes were related to the redox state, ERF, etc. of 4-day treated sample of FocTR4 infection. Eight out of ten redox state genes were identified as upregulated; moreover, in ERF, four genes were upregulated and two genes were downregulatedbut in FocTR4 Brazilian cultivar, 1-day treated with BTH, one gene was downregulated. The expression levels of four core genes were representing the secondary metabolites, of which two were expressed in ABA and JA each and one in beta-glucanase (Table S7).

## 4. Discussion

Panama disease or Fusarium wilt disease is a destructive soil-borne disease caused by the fungi Foc, which typically attacks the roots of both susceptible and resistant banana cultivars. However, in response to infection, the xylem lumina produces tyloses, gums, or gels^4^ as a defensive mechanism, especially in resistant cultivars than in susceptible cultivars, and also prevents the systemic infection of the pseudo stem in resistant cultivars, but the pathogen colonizes susceptible cultivars ahead of these host responses.

The mechanism of Foc pathogenesis in bananas still remains a mystery and needs a long understanding. Therefore, in order to fill this lacuna, a detailed study of the elements that cause pathogenicity focused on three main cultivars of Cavendish bananas and compared them with few nonpathogenic Foc strains that colonize banana roots without causing wilt disease.^35^ Since the data on mechanism underlying host defense under fungal (FocTR4) infectious condition^6^ and how BTH influenced banana plant resistance against FocTR4 infection^19^ were already available, the data exploration on integrating omics approaches to compare the Berangan, Brazilian, and Williams of Cavendish banana cultivars under FocTR4 infections as well as BTH-treated conditions gave an insight into the gainful information which are discussed below.

The current study using publicly available transcriptome data identified more number of core genes in Berangan of the Cavendish banana among approximately 56.3% of genes that were significantly expressed. Furthermore, duplicate genes were removed from significant DEGs to the modules of WGCNA analysis and identifed core genes in correspondence to specific traits, which indicated that the reduction in downregulated core genes from 946 (BerFoc0vs4d) to 31 (BraBTH0vs1d) under BTH treatment influencing the effectiveness of chemical moiety in regulating the plants by expressing the genes responsible for cell wall organization or biogenesis and external encapsulating structure organization processes. Furthermore, cellular responses to oxygen-containing compound and jasmonic acid (JA)-mediated signaling pathway were modulated as evidenced in.^19^ Our study has also revealed that Berangan cultivar genes have more influence in 4 days of Foc infection (Figure 2), resulting in 28.04% DEGs responsible for leaf yellowing and stunting when compared to others cultivars with or without BTH treatment.

In many plants such as cucumber, tobacco, wheat, and so on, it was proved that BTH treatment has provided resistance against multiple pathogens namely, *Colletotrichum lagenarium,*^36^ *Tobacco mosaic virus* (TMV), and powdery mildew^37^ infections, respectively. In the Williams cultivar, at days 2 and 4 of normal growth conditions, fewer number of genes were expressed when compared with the Berangan cultivar at day 4 of FocTR4 infection. This clearly implies that the plant shows more resistance when infected by the FocTR4, which leads more number of genes to get influenced. Furthermore, in Williams cultivar, we found that genes were linearly influenced by days^22^ as BPs like photosynthesis, regulations of biosynthesis process, and response to radiations were found more in 2-day-old plants compared to 4-day-old plants of Williams cultivar.

In WGCNA analysis, 13,989 genes across 16 samples were constructed, of which five modules were positively expressed in four samples emphasizing high stability, while two modules consisting of 1110 genes were negatively expressed in two samples. This shows that module genes were linked to the most significant DEGs shared by relevant specific samples; furthermore, it was identified that GO, biological regulation, resistance genes, cell wall organization, and protein phosphorylation processes were differentially expressed upon Foc infection in BerFoc0vs4d, which was also reported in a study by the authors.^31^ Pathway analysis was also identified under significant enrichment FDR with the help of ShinyGO v0.75^32^ tool, in which 79 genes were involved in top 20 pathways. Similarly, in BerFoc0vs4d, BraBTH0vs1d, and BraBTH0vs3d samples, significant fold enrichment of genes observed could have been associated with primary defense line, perhaps the cell-wall-mediated responses of plants. The genes were related to hemicellulose metabolic process, cell wall polysaccharide metabolic process, cell wall macromolecule metabolic process, cell wall organization, cell wall organization or biogenesis, and external encapsulating structure organization biogenesis, which were significantly enriched in the former sample. Moreover, when Brazilian cultivar was treated with BTH, fold enrichment gradually declined in the processes relating to the decrease in the expression associated with cell wall. This clearly depicted the downregulation of certain DEGs because they were involved in protection from the fungal attack through cell wall regeneration or its maintenance upon treatment.^19^ Interestingly, when BerFoc0vs4d was compared with WilNor0vs2d and WilNor0vs4d, a decrease in significant fold enrichment was visualized for the DEGs associated with the phenomenon of light harvesting and photosynthesis, suggesting an effect during the Foc infection; these genes were highly enhanced, which were not seen in the other two samples grown at normal conditions, as yellowing of the leaves and wilting are the major symptoms caused by the pathogen.^38^

All modules of WGCNA were regarded as core genes, 573 string proteins were found from 2077 cores genes, and further, PPI analysis of many genes having intersectional relations were performed. In our study we found that alpha-linolenic acid metabolism pathway was affected by FocTR4 fungus in Berangan and Brazilian cultivars, and similar reports have also been encountered in Tianbaojiao banana cultivar.^39,40^ The branched-chain amino acid transaminases play an important role in the metabolism of leucine, isoleucine, and valine,^41^ and their involvement in the biosynthesis of the amino acid (AA) pathways seemed to have been negatively influenced by the fungal infections in the banana root, which perhaps indicates that with certain enzymes catalyzing the final step of synthesis or the first step of degradation of these AAs could have been affected as reported in.^42,43^ Another protein, glyceraldehyde-3-phosphate dehydrogenases, was also found in many plants’ metabolic pathways of glycolysis that generates intermediate products for the primary metabolites such as AAs and fatty acids.^44–46^ 31 proteins were involved in the biosynthesis of secondary metabolites pathways (Table S6P), three proteins were positively expressed out of 31 proteins in the same fungi affected Brazilian cultivar and similar results were also reported in dwarf and wild-type bananas and Aifen No. 1 cultivars.^47,48^

MapMan-based analysis confirmed that the redox state of the pectinesterase (PE) gene was upregulated in Berangan cultivars under FocTR4 infections but was not significant in the Brazilian cultivar due to the the fact that more number of genes involved are responsible for PE protein metabolism apparently regulated the cell wall modification, cell adhesion, and stem elongation in dicot plants (Table S7).^49,50^ During biotic stress, it was found that in different cultivars of banana, the gene expression values of many were changed,^51,52^ and this behavioral pattern was in concordance to our study as well, wherein the peroxisome pathway playing an important role in the biosynthesis salicylic acid was downregulated in Berangan root upon FocTR4 infection as biosynthesis of salicylic acid was involved in defending the mechanism in plants.^53^ Plant hormone signal transduction pathways incorporate JA genes essential for the development of plants, especially during drought stress conditions in tomatoes^54^ infected with FocTR4, which have also been negatively regulated in Berangan cultivar roots unlike the cultivar of Brazilian roots that was on par with the studies in tomatoes being influenced under the downregulated condition. Similarly, other genes responsible for hormonal regulation such as ABA, JA, phytochromes, and TIFY proteins seemed to be triggered, and this has been in contradiction to the view explained in *Arabidopsis thaliana* as both abiotic and biotic stresses were influencing the behavior pattern of the genes differently.^55^ This variation was also evidenced during the temperature stress when ubiquitin-mediated proteolysis pathway was found to have positively influenced green ripping of bananas in association with E3 ubiquitin-ligase,^56^ but with fungal FocTR4 infection, a biotic stress, the genes have an impact in Berangan cultivar of the Cavendish bananas Schneider and Knuesting.

## 5. Conclusion

The existing transcriptome information produced by^19^ the SRA dataset of different cultivars exposed to infection and chemical moiety treatment were subjected to be compared and analyzed in order to observe the responses of genes and their products in terms of their regulatory differences in varied time intervals. From the analyses, interestingly, few genes corresponding to the basic growth like cell wall organization or biogenesis seemed to be triggered in the Berangan cultivar unlike the Brazilian cultivar of the Cavendish banana. Subsequently, gene products associated with alpha-linolenic acid metabolism pathway, BCATs, were also affected upon FocTR4 infection in Berangan and Brazilian cultivars. Based on a comparison investigation employing statistical estimations such as the DEG, 12 DEGs were found in all cultivars (Berangan, Brazilian, and Williams) under fungal infection as well as with treatment. The up- and downregulated genes when compared to the genes in the modules of WGCNA with relevant specific samples (traits), the core genes in modules blue, green, greenyellow, lightgreen, and turquoise have been identified to be upregulated and 125 genes were downregulated. The present study also reveals that many pathways have been negatively influenced in Berangan cultivar of Cavendish bananas upon FocTR4 infections and the genes influenced also increased over time in Williams cultivar. Furthermore, our study also found the elevated immune stimulus and trigger in resistance genes, photosynthesis, cell wall construction, pectin metabolism, and auxin response in the roots of the Cavendish banana.^1,48^

## Supplementary Material

Supplemental Material

## Data Availability

The raw data supporting the conclusions of this article will be made available by the authors without undue reservation to any qualified researcher.
