# Supplementary material for: Comparative transcriptomic and weighted gene co-expression network analysis to identify the core genes in the cultivars of Musa acuminata under both infected and chemical perturbated conditions
Source: Plant Signal Behav. 2023 Nov 10;18(1):2269675. doi: 10.1080/15592324.2023.2269675 (PMC10653623; doi:10.1080/15592324.2023.2269675)
Supplement: Supplemental Material [file KPSB_A_2269675_SM8365.zip › Table S1.docx]

**Table S1A** Data information

| Sample Name | Run Id | Project Id | Cultivar | Treatment | hour | day |
| --- | --- | --- | --- | --- | --- | --- |
| BerFoc0d_1 | SRR2132798 | PRJNA287860 | Berangan | nfectedFocTr4_2-48-96 | 2 | 0 |
| BerFoc0d_2 | SRR2132799 | PRJNA287860 | Berangan | nfectedFocTr4_2-48-96 | 2 | 0 |
| BerFoc4d_1 | SRR3727682 | PRJNA322439 | Berangan | infectedFocTr4-2-48-96 | 96 | 4 |
| BerFoc4d_2 | SRR3727687 | PRJNA322439 | Berangan | infectedFocTr4-2-48-96 | 96 | 4 |
| BraBTH0d_1 | SRR6311410 | PRJNA417328 | Brazilian | InfFocTr4-with-BTH-0-24-72 | 0 | 0 |
| BraBTH0d_2 | SRR6311412 | PRJNA417328 | Brazilian | InfFocTr4-with-BTH-0-24-72 | 0 | 0 |
| BraBTH1d_1 | SRR6311407 | PRJNA417328 | Brazilian | InfFocTr4-with-BTH-0-24-72 | 24 | 1 |
| BraBTH1d_2 | SRR6311408 | PRJNA417328 | Brazilian | InfFocTr4-with-BTH-0-24-72 | 24 | 1 |
| BraBTH3d_1 | SRR6311404 | PRJNA417328 | Brazilian | InfFocTr4-with-BTH-0-24-72 | 72 | 3 |
| BraBTH3d_2 | SRR6311406 | PRJNA417328 | Brazilian | InfFocTr4-with-BTH-0-24-72 | 72 | 3 |
| WilNor0d_1 | SRR3406511 | PRJNA319058 | Williams | Normal | 1 | 0 |
| WilNor0d_2 | SRR3406512 | PRJNA319058 | Williams | Normal | 1 | 0 |
| WilNor2d_1 | SRR3406514 | PRJNA319058 | Williams | Normal | 48 | 2 |
| WilNor2d_2 | SRR3406515 | PRJNA319058 | Williams | Normal | 48 | 2 |
| WilNor4d_1 | SRR3406517 | PRJNA319058 | Williams | Normal | 96 | 4 |
| WilNor4d_2 | SRR3406518 | PRJNA319058 | Williams | Normal | 96 | 4 |

**Table S1B** Experimental Design to identify the common genes in different combinations of data.

| Sample Replicate | Control |  | Sample replicate | Treated | DEGs |
| --- | --- | --- | --- | --- | --- |
| BerFoc0d_1 | BerFoc0d | Vs | BerFoc4d | BerFoc4d_1 | BerFoc0vs4d |
| BerFoc0d_2 |  |  |  | BerFoc4d_2 |  |
| BraBTH0d_1 | BraBTH0d | Vs | BraBTH1d | BraBTH1d_1 | BraBTH0vs1d |
| BraBTH0d_2 |  |  |  | BraBTH1d_2 |  |
| BraBTH0d_1 |  | Vs | BraBTH3d | BraBTH3d_1 | BraBTH0vs3d |
| BraBTH0d_2 |  |  |  | BraBTH3d_2 |  |
| WilNor0d_1 | WilNor0d | Vs | WilNor2d | WilNor2d_1 | WilNor0vs2d |
| WilNor0d_2 |  |  |  | WilNor2d_2 |  |
| WilNor0d_1 |  | Vs | WilNor4d | WilNor4d_1 | WilNor0vs4d |
| WilNor0d_2 |  |  |  | WilNor4d_2 |  |
|  |  |  |  |  |  |

BerFoc0d: control sample of Berangan; BerFoc4d: *Fusarium oxysporum* *f. sp. cubense* tropical race 4 (FocTR4) four days treated; BraBTH0d: *Fusarium oxysporum* *f. sp. cubense* tropical race 4 (FocTR4) zero day treated; BraBTH1d: *Fusarium oxysporum* *f. sp. cubense* tropical race 4 (FocTR4) one day treated; BraBTH3d: *Fusarium oxysporum* *f. sp. cubense* tropical race 4 (FocTR4) three day treated; WilNor0d: control sample of Williams cultivar; WilNor2d: Two days old normal sample of Williams cultivar; WilNor4d: Two days old normal sample of Williams cultivar.
